# Molecular modeling of lipid drug formulations

**DOI:** 10.1186/1758-2946-4-S1-O23

**Published:** 2012-05-01

**Authors:** Woldeamanuel A Birru, Dallas B Warren, Christopher JH Porter, Colin W Pouton, David K Chalmers

**Affiliations:** 1 Monash Institute of Pharmaceutical Sciences, Monash University, 381 Royal Pde, Parkville, VIC, Australia

## 

Lipid formulations can improve the bioavailability of drugs that have low aqueous solubility. A variety of chemical compounds, including triglyceride oils (lipids), fatty acid esters and surfactants, can be included in lipid formulations. This heterogeneity makes spectroscopic study of the in ternal structure of formulation difficult. Understanding of lipid formulations at a molecular level will greatly improve our knowledge of in vivo dispersion and solubilisation patterns of lipid formulations.

**Figure 1:**
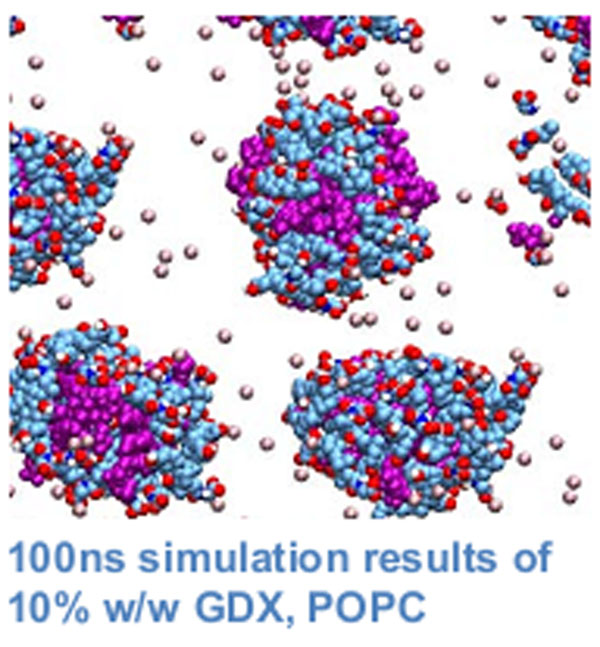


Molecular dynamics studies have provided useful insight into the structure and dynamics of different types of aggregates, including mixed glycerides with and without propylene glycol [1] and bile salts [2]. To date, such studies have not been performed on lipid drug formulations . The objective of this research is to develop a molecular dynamics protocol to examine the interaction between drugs and formulations at the atomic level. To evaluate and parameterize the force field of choice we are calculating Gibbs free energy of solvation of a number of alcohols and short poly -(ethylene glycol) polymers. Following this, the aggregation behaviour of 1-palmitoyl-2-oleoyl-sn-glycero-3-phosphocholine (POPC), sodium glycochenodeoxycholate (GDX), different digestion products and polyethylene glycol surfactants will be investigated. Moreover, the phase diagram s of three component systems composed of i) bile salts, digested products and water and ii) surfactants, lipids and water will be modelled.

Simulations are being performed using the molecular dynamics software suite GROMACS. Calculations are being performed on a high performance computing cluster at the Victorian Life Sciences Com putation Initiative (VLSCI). The methods highlighted in this study will prove to be an essential tool for formulators of lipid systems for oral administration.
